# Supplementary material for: Molecular architecture underlying fluid absorption by the developing inner ear
Source: eLife. 2017 Oct 10;6:e26851. doi: 10.7554/eLife.26851 (PMC5634787; doi:10.7554/eLife.26851)
Supplement: Figure 2—source data 1. [file elife-26851-fig2-data1.docx]

Figure 2 – Source Data 1. Summary of numbers of cells captured and sequenced

|  | Age | Samples sequenced | | Cell capture results^a^ | | | Outliers^b^ | Single-cells analyzed |
| --- | --- | --- | --- | --- | --- | --- | --- | --- |
|  |  | (/chip) | (/age) | Single-cells | Double-cells | Undetermined |  |  |
| Isolation 1 | E12.5 | 84 | 84 | 46 | 38 | 0 | 2 | 44 |
| Isolation 2 | E16.5 | 60 | 127 | 49 | 43 | 35 | 8 | 41 |
| Isolation 3 | E16.5 | 67 |  |  |  |  |  |  |
| Isolation 4 | P5 | 41 | 125 | 92 | 25 | 8 | 10 | 82 |
| Isolation 5 | P5 | 43 |  |  |  |  |  |  |
| Isolation 6 | P5 | 41 |  |  |  |  |  |  |
| Isolation 7 | P30 | 55 | 96 | 54 | 20 | 22 | 8 | 46 |
| Isolation 8 | P30 | 41 |  |  |  |  |  |  |
|  | **Total** |  |  |  |  |  |  | **213** |

^a^After receiving a recent report from the manufacturer (Fluidigm) that the medium-size microfluidic chips showed an unexpected high rate of doublets (two cells in one capture site), we re-evaluated z-stack images of sequenced cells and excluded all potential doublets and undetermined samples.

^b^Outliers were identified using the SINGuLAR Analysis Toolset 3.0 (Fluidigm).
