# Supplementary material for: Molecular architecture underlying fluid absorption by the developing inner ear
Source: eLife. 2017 Oct 10;6:e26851. doi: 10.7554/eLife.26851 (PMC5634787; doi:10.7554/eLife.26851)
Supplement: Figure 2—source data 3. [file elife-26851-fig2-data3.docx]

Figure 2 – Source Data 3: List of TaqMan® gene expression assays

| **Gene Symbol** | **TaqMan assay ID** |
| --- | --- |
| *Slc26a4* | Mm01258316_m1 |
| *Foxi1* | Mm00458451_m1 |
| *Atp6v1b1* | Mm00460309_m1 |
| *Atp6v0a4* | Mm00459882_m1 |
| *Jag1* | Mm00496902_m1 |
| *Coch* | Mm00483355_m1 |
| *Atp6v0d2* | Mm01222963_m1 |
| *Atp6v1c2* | Mm00505047_m1 |
| *Atp6v1g3* | Mm00616840_m1 |
| *Bsnd* | Mm00475532_m1 |
| *Car12* | Mm00724225_m1 |
| *Clcnkb* | Mm00490564_m1 |
| *Insrr* | Mm00442243_m1 |
| *Kcnma1* | Mm01268569_m1 |
| *Slc34a2* | Mm01215846_m1 |
| *Slc4a9* | Mm01130729_m1 |
| *Tfcp2l1* | Mm00470119_m1 |
| *Tmem213* | Mm01165265_g1 |
| *Notch1* | Mm00435249_m1 |
| *Agt* | Mm00599662_m1 |
| *AU021092* | Mm01344990_m1 |
| *Bmp3* | Mm00557790_m1 |
| *Bmp7* | Mm00432102_m1 |
| *Col11a1* | Mm00483387_m1 |
| *Col11a2* | Mm00487046_m1 |
| *Dmkn* | Mm01176534_m1 |
| *Fmod* | Mm01294874_m1 |
| *Gja1* | Mm00439105_m1 |
| *Lbp* | Mm00493139_m1 |
| *Lcn2* | Mm01324470_m1 |
| *Lect1* | Mm00495291_m1 |
| *Nox3* | Mm01339132_m1 |
