# Supplementary material for: Molecular architecture underlying fluid absorption by the developing inner ear
Source: eLife. 2017 Oct 10;6:e26851. doi: 10.7554/eLife.26851 (PMC5634787; doi:10.7554/eLife.26851)
Supplement: Figure 8—source data 1. [file elife-26851-fig8-data1.docx]

| **Chip** | **Age** | **Genotype** | **Total RNA (ng)** | **RIN** | **Scale Factor** | **Average Call All** | **%Present Call** | **Back Intensity** | **Noise** | **Raw Q** | ***Actb* 3’/5’** | ***Gapdh* 3’/5’** |
| --- | --- | --- | --- | --- | --- | --- | --- | --- | --- | --- | --- | --- |
| 1 | E13.5 | *Slc26a4*^Δ/Δ^ | 3.5 | 9.7 | 1.3 | 643 | 68.0 | 40.4 | 1.9 | 1.5 | 0.93 | 1.49 |
| 2 | E14.5 | *Slc26a4*^Δ/Δ^ | 3.4 | 9.6 | 1.0 | 608 | 70.4 | 43.3 | 2.3 | 1.7 | 0.96 | 1.53 |
| 3 | E16.5 | *Slc26a4*^Δ/Δ^ | 5.2 | 9.6 | 1.2 | 632 | 69.2 | 45.1 | 2.1 | 1.6 | 0.98 | 1.36 |
| 4 | E17.5 | *Slc26a4*^Δ/Δ^ | 4.6 | n/a | 1.5 | 650 | 69.1 | 45.9 | 2.0 | 1.7 | 0.95 | 1.36 |
| 5 | E13.5 | *Slc26a4*^Δ/+^ | 3.5 | 9.6 | 1.2 | 618 | 72.2 | 44.2 | 1.9 | 1.6 | 0.96 | 1.60 |
| 6 | E14.5 | *Slc26a4*^Δ/+^ | 3.5 | 8.8 | 1.2 | 622 | 69.7 | 38.5 | 1.9 | 1.5 | 1.03 | 1.53 |
| 7 | E16.5 | *Slc26a4*^Δ/+^ | 5.1 | 9.4 | 1.3 | 624 | 67.4 | 39.1 | 1.8 | 1.5 | 1.00 | 1.36 |
| 8 | E17.5 | *Slc26a4*^Δ/+^ | 4.6 | 9.6 | 1.0 | 615 | 64.7 | 41.3 | 1.9 | 1.5 | 1.26 | 1.69 |
| 9 | E13.5 | *Slc26a4*^Δ/Δ^ | 4.0 | 9.0 | 1.5 | 630 | 65.9 | 44.6 | 2.7 | 1.7 | 0.90 | 1.48 |
| 10 | E14.5 | *Slc26a4*^Δ/Δ^ | 4.0 | 9.1 | 1.2 | 614 | 70.0 | 39.8 | 2.3 | 1.6 | 1.16 | 2.30 |
| 11 | E16.5 | *Slc26a4*^Δ/Δ^ | 5.6 | 9.1 | 1.5 | 636 | 65.0 | 46.5 | 2.4 | 1.7 | 0.95 | 1.44 |
| 12 | E17.5 | *Slc26a4*^Δ/Δ^ | 5.8 | 9.6 | 1.4 | 629 | 64.6 | 47.7 | 2.7 | 1.8 | 1.00 | 1.40 |
| 13 | E13.5 | *Slc26a4*^Δ/+^ | 4.1 | 9.2 | 1.6 | 627 | 67.5 | 45.3 | 2.3 | 1.6 | 0.90 | 1.33 |
| 14 | E14.5 | *Slc26a4*^Δ/+^ | 4.0 | 8.7 | 1.9 | 652 | 66.4 | 51.2 | 2.3 | 1.8 | 0.93 | 1.51 |
| 15 | E16.5 | *Slc26a4*^Δ/+^ | 5.6 | 9.2 | 1.5 | 637 | 66.0 | 44.8 | 2.2 | 1.6 | 0.92 | 1.28 |
| 16 | E17.5 | *Slc26a4*^Δ/+^ | 5.9 | 9.6 | 1.5 | 635 | 64.3 | 44.8 | 2.3 | 1.7 | 1.96 | 1.32 |
| 17 | E13.5 | *Slc26a4*^Δ/Δ^ | 4.0 | 9.7 | 1.4 | 641 | 67.8 | 43.0 | 1.9 | 1.5 | 1.06 | 1.41 |
| 18 | E14.5 | *Slc26a4*^Δ/Δ^ | 4.0 | 9.3 | 1.7 | 639 | 68.1 | 41.5 | 1.7 | 1.5 | 1.12 | 1.62 |
| 19 | E16.5 | *Slc26a4*^Δ/Δ^ | 6.0 | 9.2 | 1.1 | 612 | 71.4 | 46.6 | 2.0 | 1.8 | 1.06 | 1.29 |
| 20 | E17.5 | *Slc26a4*^Δ/Δ^ | 6.0 | 9.4 | 1.4 | 624 | 67.7 | 44.4 | 2.6 | 1.7 | 0.99 | 1.29 |
| 21 | E13.5 | *Slc26a4*^Δ/+^ | 4.0 | 9.8 | 1.3 | 622 | 69.6 | 42.3 | 1.9 | 1.6 | 1.01 | 1.35 |
| 22 | E14.5 | *Slc26a4*^Δ/+^ | 4.0 | 8.7 | 1.9 | 642 | 70.1 | 39.3 | 1.7 | 1.5 | 0.90 | 1.51 |
| 23 | E16.5 | *Slc26a4*^Δ/+^ | 6.0 | 8.8 | 1.6 | 633 | 68.2 | 39.3 | 1.8 | 1.5 | 0.91 | 1.29 |
| 24 | E17.5 | *Slc26a4*^Δ/+^ | 6.0 | 9.6 | 1.7 | 641 | 67.9 | 40.0 | 1.8 | 1.5 | 0.95 | 1.21 |

Figure 8 – Source Data 1: Quality and quantity of total RNA and quality metrics of gene array chips
