# Supplementary material for: Molecular architecture underlying fluid absorption by the developing inner ear
Source: eLife. 2017 Oct 10;6:e26851. doi: 10.7554/eLife.26851 (PMC5634787; doi:10.7554/eLife.26851)
Supplement: Figure 9—source data 1. [file elife-26851-fig9-data1.docx]

Figure 9 – Source Data 1: Summary of immunolocalization of proteins in mitochondria-rich cells

| Protein | Cell Type | Subcellular localization | Age | Species | References |
| --- | --- | --- | --- | --- | --- |
| SLC26A4 | MRCs and subset of RRCs | Apical | E13.5 – adult | Mouse | Present study, 17, 18, 36 |
| ATP6V1B1 | MRCs | Apical | E16.5, E19, P5 | Mouse | Present study, 3, 23 |
| ATP6V0A4 | MRCs | Apical | E16.5, Adult | Mouse | 22, 23 |
| SLC4A9 | MRCs | Basal | P5 | Mouse | Present study |
| BSND | MRCs | Basal | P5 | Mouse | Present study |
| SCNN1A | Unknown | Apical | Adult | Human | 46 |
| SCNN1B | Unknown | Apical | Adult | Human | 46 |
| SCNN1G | Unknown | Apical | Adult | Human | 46 |
| ATP1A1 | MRCs and RRCs | Basal | E15.5 | Mouse | Present study |
