# Supplementary material for: Molecular architecture underlying fluid absorption by the developing inner ear
Source: eLife. 2017 Oct 10;6:e26851. doi: 10.7554/eLife.26851 (PMC5634787; doi:10.7554/eLife.26851)
Supplement: Transparent reporting form [file elife-26851-transrepform.docx]

***eLife’s* transparent reporting form**

We encourage authors to provide detailed information *within their submission* to facilitate the interpretation and replication of experiments. If you have any questions, please contact us: [editorial@elifesciences.org](mailto:editorial@elifesciences.org).

**Sample-size estimation**

- You should state whether an appropriate sample size was computed when the study was being designed
- You should state the statistical method of sample size computation and any required assumptions
- If no explicit power analysis was used, you should describe how you decided what sample (replicate) size (number) to use

Please outline where this information can be found within the submission (e.g., page numbers or figure legends), or explain why this information doesn’t apply to your submission:

(Rate of absorption) Grubbs’ outlier test was performed and one outlier was removed from the dataset. Normal distribution and equal variance of the data was evaluated by the Shapiro-Wilk test and Brown-Forsythe test. Statistical power was analyzed and Cohen’s d was calculated. Information is given in the Results section of the manuscript and in the Source Data for Figure 1.

(Single-cell RNA-seq) We did not attempt to calculate an appropriate sample size (number of cells) because the sample size for the RNA-seq experiment depends upon the cellular heterogeneity of the tissue. We chose up to 100 cells for each time point since our purpose was not to detect a rare population.

(Gene array) We did not attempt to calculate an appropriate sample size because this experiment is not a hypothesis-driven analysis.

**Replicates**

- You should report how often each experiment was performed
- You should include a definition of biological versus technical replication
- The data obtained should be provided and sufficient information should be provided to indicate the number of independent biological and/or technical replicates
- If you encountered any outliers, you should describe how these were handled
- Criteria for exclusion/inclusion of data should be clearly stated
- High-throughput sequence data should be uploaded before submission, with a private link for reviewers provided (these are available from both GEO and ArrayExpress)

Please outline where this information can be found within the submission (e.g., page numbers or figure legends), or explain why this information doesn’t apply to your submission:

Summary of number of cells captured and sequenced was shown in Figure2 – Source Data 1. Detailed information of each sample of single cell RNA-seq is deposited as a sample list in the GEO database. Outlier identification for single-cell RNA-seq is described at page 32 and Figure 2 – source data 2. Outlier identification for gene-array analysis is described at page 36 and figure 8 – figure supplement 1. RNA-seq data and gene-array data have been deposited in the NCBI GEO database under accession code GSE87293 and GSE90092, respectively.

**Statistical reporting**

- Statistical analysis methods should be described and justified
- Raw data should be presented in figures whenever informative to do so (typically when N per group is less than 10)
- For each experiment, you should identify the statistical tests used, exact values of N, definitions of center, methods of multiple test correction, and dispersion and precision measures (e.g., mean, median, SD, SEM, confidence intervals; and, for the major substantive results, a measure of effect size (e.g., Pearson's r, Cohen's d)
- Report exact p-values wherever possible alongside the summary statistics and 95% confidence intervals. These should be reported for all key questions and not only when the p-value is less than 0.05.

(Rate of absorption) Power and significance were evaluated by one-way ANOVA followed by an all pairwise multiple comparison procedure (Bonferroni t-test) (described at page 28).

(Single cell RNA-seq) Principal component analysis and hierarchical clustering were used to identify cell populations (described at legends of figure 2, 3, and 4). Generalized additive model was used to identify differentially expressed genes (described at pages 33 and references).

(Gene-array) A regression model and hierarchical clustering were used to identify differentially expressed genes (described at legends of figure 8).

Please outline where this information can be found within the submission (e.g., page numbers or figure legends), or explain why this information doesn’t apply to your submission:

(For large datasets, or papers with a very large number of statistical tests, you may upload a single table file with tests, Ns, etc., with reference to page numbers in the manuscript.)

**Additional data files (“source data”)**

- We encourage you to upload relevant additional data files, such as numerical data that are represented as a graph in a figure, or as a summary table
- Where provided, these should be in the most useful format, and they can be uploaded as “Source data” files linked to a main figure or table
- Include model definition files including the full list of parameters used
- Include code used for data analysis (e.g., R, MatLab)
- Avoid stating that data files are “available upon request”

Please indicate the figures or tables for which source data files have been provided:

Figure 1 – Source Data for the rate of fluid absorption

Figure 2 – Source Data 1. Summary of numbers of cells captured and sequenced

Figure 2 – Source Data 2. Cell-type specific genes identified by differential expression analysis

Figure 2 – Source Data 3. List of TaqMan® gene expression assays

Figure 8 – Source Data 1. Quality quantity of total RNA and quality metrics of gene array chips

Figure 8 – Source Data 2. Gene array analysis of embryonic endolymphatic sac in *Slc26a4*^Δ/+^ and *Slc26a4*^Δ/Δ^ mice

RNA-seq data and gene-array data have been deposited in the NCBI GEO database under accession code GSE87293 and GSE90092, respectively. We uploaded the source code for R as a text file.
